# Optimal two-stage design of single arm Phase II clinical trials based on median event time test

**DOI:** 10.1371/journal.pone.0246448

**Published:** 2021-02-08

**Authors:** Yeonhee Park

**Affiliations:** Department of Biostatistics and Medical Informatics, University of Wisconsin, Madison, WI, United States of America; Roswell Park Cancer Institute, UNITED STATES

## Abstract

The Phase II clinical trials aim to assess the therapeutic efficacy of a new drug. The therapeutic efficacy has been often quantified by response rate such as overall response rate or survival probability in the Phase II setting. However, there is a strong desire to use survival time, which is the gold standard endpoint for the confirmatory Phase III study, when investigators set the primary objective of the Phase II study and test hypotheses based on the median survivals. We propose a method for median event time test to provide the sample size calculation and decision rule of testing. The decision rule is simple and straightforward in that it compares the observed median event time to the identified threshold. Moreover, it is extended to optimal two-stage design for practice, which extends the idea of Simon’s optimal two-stage design for survival endpoint. We investigate the performance of the proposed methods through simulation studies. The proposed methods are applied to redesign a trial based on median event time for trial illustration, and practical strategies are given for application of proposed methods.

## Introduction

The primary objective of Phase II clinical trials is to test whether the therapeutic intervention actually works in treating a disease or indication. The therapeutic efficacy has been often quantified by response rate in Phase II settings, for example, overall response rate defined as the proportion of subjects who achieve a confirmed complete response or partial response determined by RECIST 1.1 [1] or survival probability at a certain year defined as the proportion of patients alive (and without recurrence) at certain year after the start of treatment. In practice, Simon’s two-stage minimax and optimal designs are widely used for binary endpoint [2–7]. Simon’s designs allow early trial termination due to futility while Fleming’s design allows early trial termination due to futility and superiority in Phase II trials [8, 9]. Successful results in Phase II trials lead to proceed to confirmatory Phase III trials with more extensive development. Therefore, it is necessary to validate and use the surrogate endpoint for overall survival, which is the gold standard endpoint for Phase III study. When considering the survival probability which enforces the survival time to the binary endpoint, we need much caution to analyze the results. Some designs address incomplete follow-up for some subjects by the time of the interim analysis to assess the survival probability [10–13]. Data augmentation can be used to address the timing of events for late-onset outcomes by imputing missing outcomes. [14, 15].

Surprisingly, a recent FDA report shows 22 case studies investigating disagreement in the results between early and confirmatory phases [16]. Some cases showed unexpected failures in Phase III study from the promising results on clinical outcomes in the Phase II study (e.g., iniparib). Therefore, some investigators strongly desire to use time-to-event endpoint to evaluate the therapeutic efficacy of the drug for Phase II trials. They are interested in improvement of median survival compared to standard therapy. Finkelstein et al. [17], Sun et al. [18], Jung [19], Kwak and Jung [20], Wu et al. [21] propose designs with one-sample log-rank test which compares the survival distributions between prespecified null reference and the desired target. This idea comparing the survival distributions allows to conduct for hypothesis testing for the median survivals only when the survivals are assumed to follow exponential distribution. Wu [22] proposes a single-arm Phase II clinical trial design under a class of parametric cure models. Chu et al. [23] proposes the design for immunotherapy trials with random delayed treatment effect based on survival time.

In this paper, we propose new methods for a single arm Phase II study, which provide practical strategies for testing if there is an improvement of median survivals from a new drug compared to the standard therapy. The proposed median event time test uses the distribution of sample median to obtain the required sample size and threshold for the hypothesis testing at target type I and II errors. It provides the decision rule, which is very simple and straightforward, comparing the observed median survival with the identified threshold at the end of trial. This can be easily implemented with our shiny application and R codes built along with the method development. Moreover, this median event time test is extended in group sequential manner. It follows the idea of Simon’s optimal two-stage design, in that the expected sample size is minimized under the null hypothesis, but considers survival endpoint to see the improvement based on the median event time test. This allows us to monitor futility of the drug based on median survival time and stop the trial early, which makes the design more efficient and practical.

The rest of the paper is organized as follows. In Methods Section, we provide a median event time test and propose an optimal two-stage design based on median event time test. In Results Section, simulation studies are presented, and a trial example is provided to illustrate the application of our methods. Lastly, we provide some comments in Discussion and concluding remarks in Conclusion.

## Methods

### Median event time test

Let *Y*_1_, …, *Y*_*n*_ be random variables from an exponential distribution with mean *μ*. Then, the median *ϕ* is equivalent to *μ* log 2. Motivated by single arm Phase II studies whose primary endpoint is time-to-event, we formulate a hypothesis test based on median event time with *H*_0_: *ϕ* = *ϕ*_0_ versus *H*_*a*_: *ϕ* = *ϕ*_1_ for some values of *ϕ*_0_ and *ϕ*_1_. In practice for intervention study, we use median time of standard drug or therapy and the expected median time from the intervention (for improvement) to specify the values of *ϕ*_0_ and *ϕ*_1_, respectively. Let ${\hat{\phi }}_{n}\left(y\right)$ be a sample median event time obtained from a sample of size *n*. Then, for any λ ≥ 0, the rejection region for the test is $\left\{y:{\hat{\phi }}_{n}\left(y\right)>\lambda \right\}$. For any *α* and *β* in unit interval (0, 1), this test is the level *α* test with power 1 − *β* such that $\text{Pr}\{{\hat{\phi }}_{n}\left(y\right)>\lambda |H_{0}\}=\alpha$ and $\text{Pr}\{{\hat{\phi }}_{n}\left(y\right)\leq \lambda |H_{\text{a}}\}=\beta$. Since the first conditional probability indicates the probability of rejecting the null hypothesis when the null hypothesis is true, the value of *α* indicates the type I error rate. Also, the second conditional probability indicates the probability of failure of rejecting null hypothesis when the null hypothesis is not true, and the value of *β* indicates the type II error rate. The distribution function of the sample median event time is derived in the following theoretical result.

**Theorem 1** *Let Y*_1_, …, *Y*_*n*_ *be random variables from an exponential distribution with median ϕ. Then, the probability density function (pdf) of sample median of Y*_1_, …, *Y*_*n*_ *is either*

$$\begin{array}{c} g_{n}\left(m\right)=n!\;\text{log}\;2{\left\{1-\exp\left(-m\;\text{log}\;2/\phi \right)\right\}}^{k}\exp\left\{-m\left(k+1\right)\;\text{log}\;2/\phi \right\}/\left(k!k!\phi \right) \end{array}$$

*for n* = 2*k* + 1 *or*

$$\begin{array}{ccc} g_{n}\left(m\right) & = & \int_{0}^{2m}n!{\left(\;\text{log}\;2\right)}^{2}\exp\left(-2m\left(\;\text{log}\;2\right)/\phi \right){\left\{1-\exp\left\{-\left(\;\text{log}\;2\right)\left(m-r/2\right)/\phi \right\}\right\}}^{k-1}\\ & & \times \exp\left\{-\left(\;\text{log}\;2\right)\left(m+r/2\right)\left(n-k-1\right)/\phi \right\}/\left\{\left(k-1\right)!\left(n-k-1\right)!\phi^{2}\right\}\;dr \end{array}$$

*for n* = 2*k*.

The proof is in Appendix A. Theorem 1 provides a cumulative distribution function (cdf) of median event time given by $G_{n}\left(\lambda \right)=\int_{0}^{\lambda }g_{n}\left(m\right)\;\text{d}m$, where *g*_*n*_ denotes the pdf of the sample median, for λ ≥ 0. Since the cdf of sample median event time has no closed form, a numerical search over grid is required to identify sample size *n* and threshold λ such that the empirical errors $\hat{\alpha }\left(n,\lambda \right)$ and $\hat{\beta }\left(n,\lambda \right)$ are close to the nominal target values of type I and II error rate (i.e., *α* and *β*), respectively. The empirical errors are calculated as

$$\begin{array}{c} \hat{\alpha }\left(n,\lambda \right)=\text{Pr}\left({\hat{\phi }}_{n}>\lambda |H_{0}\right)=1-G_{n}\left(\lambda |H_{0}\right)\;\;\;\text{and}\;\;\;\hat{\beta }\left(n,\lambda \right)=\text{Pr}\left({\hat{\phi }}_{n}\leq \lambda |H_{\text{a}}\right)=G_{n}\left(\lambda |H_{\text{a}}\right), \end{array}$$

where *G*_*n*_(⋅|*H*_0_) and *G*_*n*_(⋅|*H*_a_) denote the cdf of sample median of event time under null and alternative hypotheses, respectively. It implies that the identified sample size *n* justifies to achieve $\{1-\hat{\beta }\left(n,\lambda \right)\}*100$% power based on one-sided test with a significance level of $\hat{\alpha }\left(n,\lambda \right)$ to detect improvement of the sample median time of the experimental drug against the median time of standard drug. This test states that at the end of trial, i.e., based on sample of size *n*, the null hypothesis is rejected if ${\hat{\phi }}_{n}>\lambda$ and we argue that experimental drug increases in median event time from *ϕ*_0_ to *ϕ*_1_. We call this median event time test.

**Fig 1:**
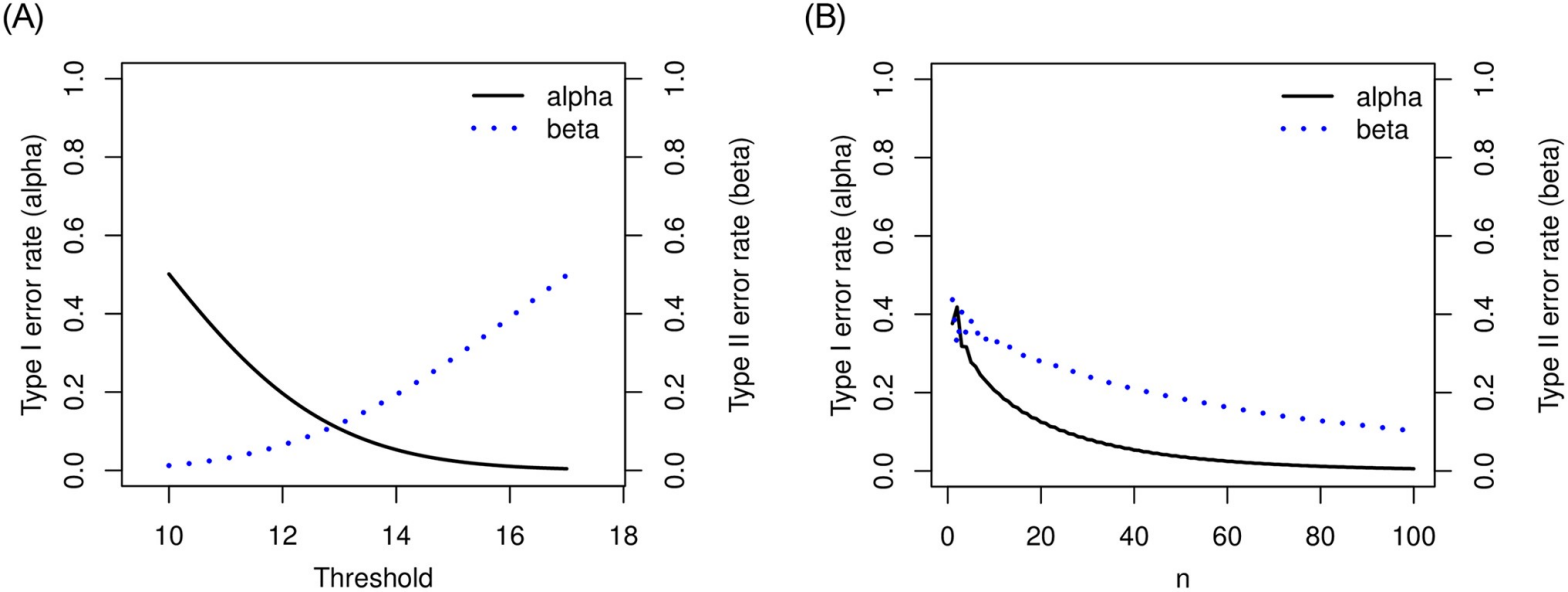
Plot of $\hat{\alpha }\left(n,\lambda \right)$ and $\hat{\beta }\left(n,\lambda \right)$ for median event time test with *ϕ*_0_ = 10 and *ϕ*_1_ = 17 months: Left panel shows the result when *n* = 42 and right panel shows the results when λ = 14.1 months.

Let’s consider a clinical trial with hypothesis testing of median progression free survivals (PFS) *ϕ*_0_ = 10 months versus *ϕ*_1_ = 17 months. Suppose that maximum number of patients for this study is 100. We consider a grid search over the integer *n* between 1 and 100 and a real number λ between 10 and 17 with the increment 0.1. For the target error rates of *α* = 0.05 and *β* = 0.2, our numerical study results in *n* = 42 and λ = 14.1 months minimizing deviation of the empirical errors from the target rates, i.e., ${\left\{\hat{\alpha }\left(n,\lambda \right)-\alpha \right\}}^{2}+{\left\{\hat{\beta }\left(n,\lambda \right)-\beta \right\}}^{2}$. Fig 1 shows results for numerical search of *n* and λ, and Table 1 summarizes the decision rule for testing median survival *ϕ*_0_ versus *ϕ*_1_. In Table 1, scenarios with different value of *ϕ*_0_ and *ϕ*_1_ are considered. The choice of *ϕ*_0_ and *ϕ*_1_ is determined intentionally. To see the performance of method, we fixed *ϕ*_0_ or *ϕ*_1_ to vary *ϕ*_1_ or *ϕ*_0_, respectively.

**Table 1:** Decision rules for hypothesis testing of median PFS *ϕ*_0_ months versus *ϕ*_1_ months at target error rates of *α* = 0.05 and *β* = 0.2. Numerical search is done over the integer *n* between 1 and 100 and a real number λ between *ϕ*_0_ and *ϕ*_1_ with the increment 0.1.

*ϕ*_0_	*ϕ*_1_	*n*	λ	$\hat{\alpha }\left(n,\lambda \right)$	$\hat{\beta }\left(n,\lambda \right)$
10	17	42	14.1	0.049	0.2019
8	17	21	12.9	0.049	0.1974
8	14	38	11.5	0.048	0.2016
3	7	16	5.2	0.042	0.2007
3	6	24	4.7	0.047	0.2027
3	5	48	4.2	0.042	0.2030

We have proposed median event time test for exponential survivals. The proposed method is extended for other parametric survival distributions. We provide the distribution function of the sample median event time for uniform and weibull survivals in Appendix B, which determines the decision rule.

This proposed median event time test is attractive with three reasons. First, this is newly proposed for an exact median event time test using the observed median event time. Second, author provides a software to calculate sample size and identify the threshold for the test, which is open for public use (https://yeonhee.shinyapps.io/METTshinyapp/). It does not require complicated statistical analysis for decision but implements easily. The simple and straightforwardly interpretable rule can save lots of time for drug development. Third, it has potential to extend for many applications. For example, the decision rule can be used to monitor futility in group sequential designs (for Phase II or III studies), and the threshold λ provides a good candidate (or range) for the Bayesian monitoring rule. Specifically, it provides foundation for optimal two-stage design based on median event time test which is described later.

Before we move to next section, we provide a remark on the median event time test. To develop the exact median event time test, we considered hypothesized values for median of survivals *Y*_*i*_ = *min*(*S*_*i*_, *U*_*i*_), where *S*_*i*_ denote the time-to-event and *U*_*i*_ denote the administrative censoring time for the *i*th patient. Our setting in this section uses *Y*_*i*_ and does not require information for accrual rate and follow-up time. However, when we consider a hypothesis testing with median of survivals *S*_*i*_, we should care the censoring information. Assume that the time to arrival of the patient and survival time (i.e., *S*_*i*_) follow some exponential distributions. Then, *U*_*i*_, which is the time to arrival of the last patient plus follow-up time minus time to arrival of the *i*th patient, follows another exponential distribution. We notice that minimum of two exponential random variables (i.e., *Y*_*i*_) follow some exponential distribution. Therefore, this setting for hypothesis testing with *Y*_*i*_ looks reasonable with the exponential survival assumption for *S*_*i*_ and the right censoring.

### Optimal two-stage design based on median event time test

The proposed median event time is straightforward for clinicians to interpret and justify the sample size for the exact level of errors. It is critical, especially in rare disease trials, to obtain promising evidence with the target error rates and minimize the expected sample size. Moreover, from an ethical and practical viewpoint, it is desirable to stop the trial early if the therapeutic intervention is not effective. This motivates to propose a single arm Phase II trial with an interim planned when the total accrual reached *n*_1_ patients. The final analysis will be performed after the follow-up of all planned number of *n* patients. A two-stage design using median event time test is proposed as follows. In the first stage (i.e., at interim), we determine go/no-go of the trial based on the observed median event time for those *n*_1_ patients. When the observed median event time is less than or equal to the threshold λ, the trial is stopped for futility. Otherwise, the trial continues to enroll (*n* − *n*_1_) patients in the second stage. At final analysis, we argue that the drug is sufficiently promising to evaluate against the standard therapy if the observed median event time based on all trial data is larger than the threshold.

The expected sample size for the two-stage design above is EN = *n*_1_ + (1 − PET)(*n* − *n*_1_), where PET denotes the probability of early termination after the first stage. EN depends on *n*, *n*_1_ and λ, and the optimal two-stage design using median event time test is proposed in that EN is minimized under the null hypothesis. In the following, we describe how to specify *n*, *n*_1_ and λ for the optimal two-stage design. Let *M* be a maximum sample size for the study, e.g., this can be specified by clinicians or by sample size calculator for one-arm nonparametric statistics (https://stattools.crab.org/Calculators/oneNonParametricSurvival.htm). Let *ϵ*_1_ and *ϵ*_2_ denote the acceptable difference between the estimated type I and II error rates from the trial design and the target error rates. For each prespecified values of *ϕ*_0_, *ϕ*_1_, *α* and *β*,

Step 1 For each *n*_1_ between 1 and *M* − 1, search *n* and λ minimizing ${\left\{\hat{\alpha }\left(n_{1},n,\lambda \right)-\alpha \right\}}^{2}+{\left\{\hat{\beta }\left(n_{1},n,\lambda \right)-\beta \right\}}^{2}$, where
  $$\begin{array}{c} \hat{\alpha }\left(n_{1},n,\lambda \right)=\text{Pr}\left({\hat{\phi }}_{n_{1}}>\lambda ,{\hat{\phi }}_{n}>\lambda |H_{0}\right) \end{array}$$ (1)
  $$\begin{array}{c} \hat{\beta }\left(n_{1},n,\lambda \right)=\text{Pr}\left({\hat{\phi }}_{n_{1}}\leq \lambda |H_{\text{a}}\right)+\text{Pr}\left({\hat{\phi }}_{n_{1}}>\lambda ,{\hat{\phi }}_{n}\leq \lambda |H_{\text{a}}\right), \end{array}$$ (2)
  over *n*_1_ < *n* ≤ *M* and *ϕ*_0_ ≤ λ ≤ *ϕ*_1_.
Step 2 Choose an optimal pair of (*n*, *n*_1_, λ) minimizing EN among the pairs satisfying $|\hat{\alpha }\left(n_{1},n,\lambda \right)-\alpha |<\epsilon_{1}$ and $|\hat{\beta }\left(n_{1},n,\lambda \right)-\beta |<\epsilon_{2}$.

As seen in median event time test, we don’t have closed forms for the marginal or joint distributions of sample median event times to calculate empirical probabilities in (1) and (2). The numerical search is used in the first step. The criteria given in the second step targets at minimizing the expected sample size, EN. This optimality criteria can be modified according to the study objective, e.g., the design minimizes expected total study length. Moreover, our design is flexible to use different thresholds, λ_1_ for the interim analysis and λ_2_ for the final analysis. Investigators fix a threshold λ_1_ to stop early for futility and search threshold λ_2_ satisfying the target error rates (or they can search both thresholds):

$$\begin{array}{c} \hat{\alpha }\left(n_{1},n,\lambda_{1},\lambda_{2}\right)=\text{Pr}\left({\hat{\phi }}_{n_{1}}>\lambda_{1},{\hat{\phi }}_{n}>\lambda_{2}|H_{0}\right)\\ \hat{\beta }\left(n_{1},n,\lambda_{1},\lambda_{2}\right)=\text{Pr}\left({\hat{\phi }}_{n_{1}}\leq \lambda_{1}|H_{\text{a}}\right)+\text{Pr}\left({\hat{\phi }}_{n_{1}}>\lambda_{1},{\hat{\phi }}_{n}\leq \lambda_{2}|H_{\text{a}}\right)\\ |\hat{\alpha }\left(n_{1},n,\lambda_{1},\lambda_{2}\right)-\alpha |<\epsilon_{1}\;\text{and}\;\left|\hat{\beta }\left(n_{1},n,\lambda_{1},\lambda_{2}\right)-\beta \right|<\epsilon_{2}. \end{array}$$

Although the proposed method does not assume specific survival distribution, the probability calculation requires to specify the survival distribution. We can borrow information from the previous research to specify the survival distribution (e.g., exponential, uniform, or weibull distribution). As an example to illustrate the optimal two-stage design based on median event time test, we assume that the survivals follow from an exponential distribution with median *ϕ*_⋅_ and will be right-censored for subjects who have not yet met the criteria at the date of the last valid disease assessment. Setting with median survivals *ϕ*_0_ = 10 and *ϕ*_1_ = 17 for the standard therapy and a new therapy, respectively, and target error rates *α* = 0.05 and *β* = 0.2, we consider the maximum allowable sample size *M* = 50 (which is obtained from a sample size calculator for one-arm nonparametric statistics). Patients arrived according to a Poisson process with the accrual rate of 1.04 patients per month. We continue follow-up for 24 months after the last patient was enrolled. Empirical estimate of type I and II errors were obtained based on 1000 simulation trials. Then, our method with *ϵ*_1_ = 0.02 and *ϵ*_2_ = 0.025 attains the optimal results at *n*_1_ = 32, *n* = 42 and λ = 15.2. It implies that our two-stage design accrue *n*_1_ = 32 patient for the first stage. At interim, if the observed median event time based on these *n*_1_ patients is less than or equal to λ = 15.2 months, the study will be early stopped for futility. Otherwise, additional *n* − *n*_1_ = 10 patients will be accrued in stage 2, resulting in a total sample size of *n* = 42. At the end of trial, if the observed median event time based on all *n* = 42 patients is larger than λ = 15.2 months, we reject the null hypothesis and claim that the treatment is sufficiently promising. Different null and alternative median event times can be considered, and the results of the optimal two-stage design are summarized in Table 2.

**Table 2:** Summary results of optimal two-stage design for hypothesis testing of median survivals *ϕ*_0_ versus *ϕ*_1_ when survivals are assumed to follow an exponential distribution. Note *M* denotes the maximum sample size for the study calculated from one-arm nonparametric statistics. Notations such as EN_0_ and PET_0_ are used to denote EN and PET, respectively, under the null hypothesis.

			*n* is searched						
*ϕ*_0_	*ϕ*_1_	*M*	*n*_1_	*n*	λ	$\hat{\alpha }$	$\hat{\beta }$	EN_0_	PET_0_
10	17	50	32	42	15.2	0.06	0.222	32.6	0.94
8	17	26	22	26	12.1	0.065	0.212	22.26	0.94
8	14	45	29	37	12.6	0.067	0.199	29.54	0.93
3	7	21	12	21	5.1	0.063	0.204	12.57	0.94
3	6	31	13	30	5	0.062	0.225	14.05	0.94
3	5	54	17	44	4.9	0.067	0.221	18.81	0.93
			*n* = *M* is prespecified						
*ϕ*_0_	*ϕ*_1_	*M*	*n*_1_		λ	$\hat{\alpha }$	$\hat{\beta }$	EN_0_	PET_0_
10	17	50	34		15.3	0.062	0.21	34.99	0.94
8	17	26	23		12.4	0.066	0.207	23.30	0.93
8	14	45	28		12.4	0.062	0.224	29.05	0.94
3	7	21	12		5.1	0.062	0.198	12.56	0.94
3	6	31	13		5	0.061	0.223	14.10	0.94
3	5	54	17		4.9	0.063	0.225	19.33	0.94

The proposed optimal two-stage design finds, in the first stage, the total sample size *n* and threshold λ for the given interim size *n*_1_ and target error rates (i.e., *α* and *β*). In other words, both *n* and λ are searched. In case where study has a certain planned total sample size, the proposed design is tailored to identify the threshold λ in the first stage for the given interim size *n*_1_, total sample size, and target error rates. In other words, only λ is searched, and *n* is prespecified. As an example, the value of *M* obtained from one-arm nonparametric statistics in Table 2 is used for the prespecified total sample size *n* in scenarios. The results are also summarized in Table 2.

Replacing *M* = 100 in Table 1 with the value obtained from one-arm nonparametric statistics, we obtained the same results. Tables 1 and 2 show that decision rule of two-stage design yields smaller expected sample size under the null than the one-stage design except the case with *ϕ*_0_ = 8 and *ϕ*_1_ = 17 requires 2 or 3 more patients to be enrolled. When *ϕ*_0_ = 3 and *ϕ*_1_ = 5, the total sample size *n* for two-stage design is smaller than one-stage design, and the expected sample size under the null is much smaller. More patients can avoid from treating the ineffective drug under the two-stage design.

## Results

**Fig 2:**
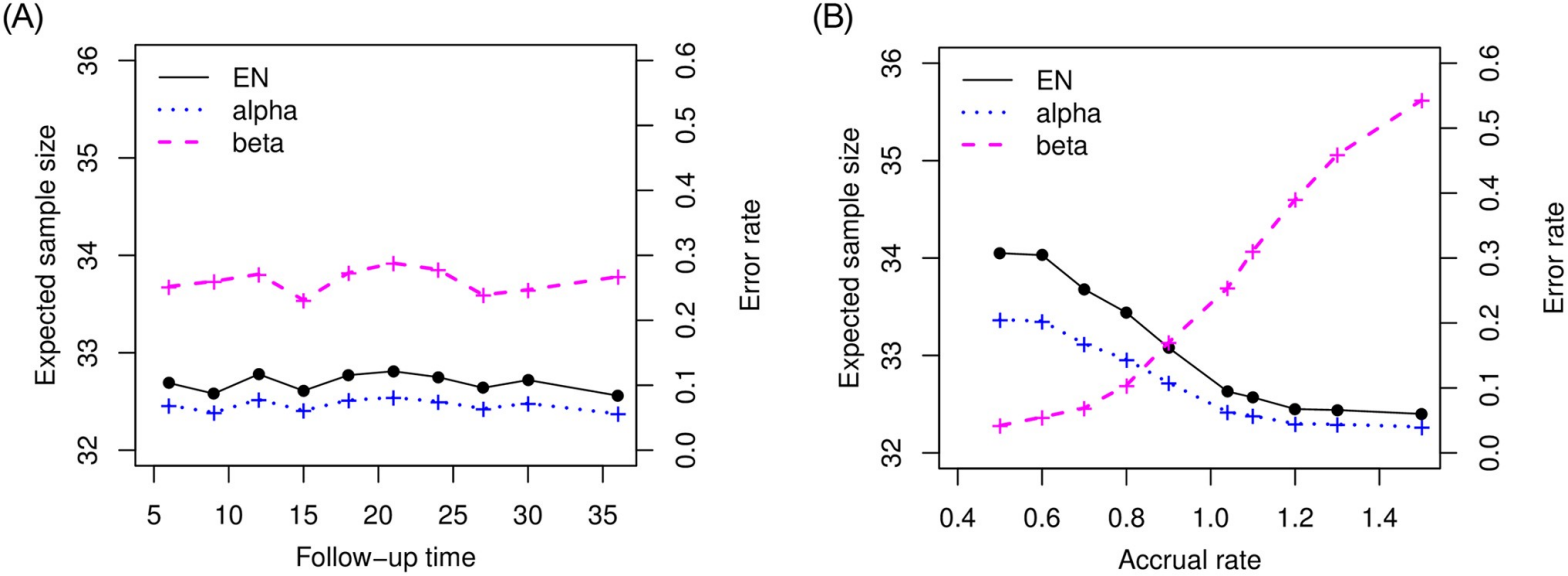
Plot of operating characteristics (i.e., expected sample size and error rates) in follow-up time and accrual rate when testing null median 10 months versus alternative median 17 months.

We investigated the performance of the proposed optimal two-stage design based on median event time test. First, back to the setting we examined for Table 2 with null median 10 months and alternative median 17 months, we are interested how the operating characteristics are changed with follow-up time and accrual rate. Given the specified rule from Table 2 (i.e., *n*_1_ = 32, *n* = 42 and λ = 15.2), we considered follow-up times 6, 9, 12, 15, 18, 21, 24, 27, 30, 36 months and accrual rates 0.5, 0.6, 0.7, 0.8, 0.9, 1.04, 1.1, 1.2, 1.3, 1.5 patients per month. The results are described in Fig 2. The proposed design is robust to the follow-up time but impacted by the accrual rate. As more patients are accrued and we have more available events (i.e., the observed median survival is less), it is more likely to stop earlier due to futility, which increases type II error rate but decrease type I error rate. Therefore, when the study is designed by using the optimal two-stage design based on median event time test, we need to have more reliable information for accrual rate, for example, the previous history for accrual rate and close collaboration with clinicians investigating the study can provide the right design.

Moreover, we examined the performance of the proposed design with non-exponential survivals. Table 3 provides the results when survivals are generated from either uniform or weibull distributions. We used uniform distribution with minimum value 0 and maximum value 2*ϕ*_⋅_ to generate flat survivals and Weibull distribution with scale parameter *ϕ*_⋅_/(log 2)^1/2^ and shape parameters 2 to obtain an increasing hazard. Compared with the results in Table 2 where survivals are assumed to follow exponential distribution, we can see the different decision rule (*n*_1_ and λ) and required sample size (*n*) for nonexponential survival times.

**Table 3:** Summary results of optimal two-stage design for hypothesis testing of median survivals *ϕ*_0_ versus *ϕ*_1_ when survivals are assumed to follow non-exponential distributions (i.e., uniform or weibull distribution). Note *M* denotes the maximum sample size for the study calculated from one-arm nonparametric statistics, and EN_0_ denotes EN under the null hypothesis.

				*n* is searched						*n* = *M* is prespecified				
	*ϕ*_0_	*ϕ*_1_	*M*	*n*_1_	*n*	λ	$\hat{\alpha }$	$\hat{\beta }$	EN_0_	*n*_1_	λ	$\hat{\alpha }$	$\hat{\beta }$	EN_0_
Uniform	10	17	50	17	50	10.9	0.052	0.211	18.72	18	11	0.061	0.197	19.95
	8	17	26	14	26	9.2	0.049	0.203	14.59	14	9.2	0.059	0.213	14.71
	8	14	45	14	42	8.7	0.064	0.206	15.79	14	8.8	0.054	0.209	15.67
	3	7	21	7	20	3.5	0.049	0.197	7.64	7	3.5	0.051	0.194	7.71
	3	6	31	7	30	3.4	0.053	0.204	8.22	7	3.4	0.053	0.2	8.27
	3	5	54	7	46	3.3	0.057	0.225	9.22	8	3.3	0.06	0.196	10.76
Weibull	10	17	50	18	21	12.1	0.047	0.205	18.14	16	11.2	0.067	0.224	18.28
	8	17	26	13	23	9.4	0.051	0.208	13.51	14	9.6	0.032	0.194	14.38
	8	14	45	14	24	9.4	0.052	0.201	14.52	14	9.1	0.067	0.199	16.08
	3	7	21	6	21	3.6	0.043	0.217	6.65	6	3.5	0.061	0.209	6.92
	3	6	31	7	16	3.7	0.052	0.196	7.47	7	3.6	0.046	0.196	8.10
	3	5	54	8	16	3.7	0.054	0.199	8.43	7	3.5	0.043	0.205	9.02

We compared the proposed two-stage designs (called METT2E, METT2U, and METT2W for optimal two-stage designs assuming exponential, uniform, and weibull survivals, respectively) with three designs: (1) a restricted KJ design, called r-KJ [12], which tests for whole survival curves based on one-sample log-rank test statistics proposed by Kwak and Jung [20]; (2) a two-stage design minimizing expected sample size, called OES [11]; (3) a two-stage design minimizing expected total study length, called OETSL [11]. Both OES and OETSL use the normalized Z-statistic to test and determine decision rules at each stage. Because r-KJ, OES, and OETSL require the clinically meaningful time point, in our simulations we used 6 months. The null and alternative values of the 6 months survival probability were determined by the survival distribution. We assumed the follow-up time is 24 months and accrual rate is 1.04 patients per month, which is the same as the setting of Tables 2 and 3. Table 4 provides the comparison results of EN_0_ and PET_0_ for several hypothesis testings based on median survival times *ϕ*_0_ and *ϕ*_1_. As seen in Tables 2 and 3, both METT2E and METT2U are likely to enroll more patients in the trial (i.e., *n* is close to *M*), compared to METT2W in most cases. The number of patients in the first stage (i.e., *n*_1_) obtained from METT2U or METT2W is smaller than that of METT2E. Thus, METT2W yields smaller expected sample size than METT2E and METT2U. Since r-KJ does not restrict to certain survival distribution and both OES and OETSL assume the weibull survivals, the results of METT2W are comparable with r-KJ, OES, and OETSL. In most cases, METT2W uses smaller expected sample size and stops the trial early for futility when therapeutic intervention is not effective. METT2E and METT2U also yielded smaller expected sample size and larger probability to stop trial for futility under the null hypothesis compared to r-KJ.

**Table 4:** Comparison results of the proposed designs (METT2E, METT2U, and METT2W) with r-KJ, OES, and OETSL. Note *M* denotes the maximum sample size for the study calculated from one-arm nonparametric statistics, EN_0_ and PET_0_ denote EN and PET, respectively, under the null hypothesis.

			EN_0_					
*ϕ*_0_	*ϕ*_1_	*M*	METT2E	METT2U	METT2W	r-KJ	OES	OETSL
10	17	50	32.6	18.72	18.14	72.38	49.08	49.00
8	17	26	22.26	14.59	13.51	34.06	25.74	25.54
8	14	45	29.54	15.79	14.52	54.52	31.52	31.38
3	7	21	12.57	7.64	6.65	12.83	7.00	6.00*
3	6	31	14.05	8.22	7.47	17.58	8.41	6.90
3	5	54	18.81	9.22	8.43	29.71	10.60	9.77
			PET_0_					
*ϕ*_0_	*ϕ*_1_	*M*	METT2E	METT2U	METT2W	r-KJ	OES	OETSL
10	17	50	0.94	0.95	0.95	0.57	0.64	0.61
8	17	26	0.94	0.95	0.95	0.56	0.69	0.62
8	14	45	0.93	0.94	0.95	0.59	0.69	0.63
3	7	21	0.94	0.95	0.96	0.54	0.76	0.94*
3	6	31	0.94	0.95	0.95	0.54	0.76	0.18
3	5	54	0.93	0.94	0.95	0.56	0.72	0.40

* Clinical time point of 5.7 months is used.

### Application: Trial illustration

We provide an application of the proposed methods with a trial NCT00780494. This is a Phase II, single arm, single-institution study of bevacizumab in combination with carboplatin and capecitabine for patients with unresectable or metastatisc gastroesophageal junction or gastric cancers. The study was started on February 2009 and completed on December 2017 to accrue enrollment of 35 participants. It enrolled two patients per month and follow up at time of study completion for 12 months. The study objective is to investigate the efficacy of the addition of bevacizumab to standard chemotherapy based on progression-free survival (PFS), which is defined as the duration of time from the start of treatment to time of disease progression or death. The median PFS is 5 months with the standard treatment therapy and the study team hypothesized the addition of bevacizumab to standard chemotherapy improve PFS by 90%, i.e., the median PFS is 9.5 months (https://clinicaltrials.gov/ProvidedDocs/94/NCT00780494/Prot_SAP_000.pdf).

We applied the proposed methods to redesign the trial based on median PFS. Target error rates are *α* = 0.05 and *β* = 0.2, and the maximum sample size is 35 obtained from one-arm nonparametric test. First, we considered a case where investigators want a trial without interim monitoring to collect the data with all 35 patients. The exact median event time test was applied, and provided the decision rule with threshold 7.5 months for a sample of size 29. This yielded 80.48% power. Second, we supposed that investigators want an interim for futility monitoring. Assuming the exponential survivals, we applied optimal two-stage design based on median event time test. We set *ϵ*_1_ = *ϵ*_2_ = 0.005 to get closer empirical error rates to the target rates, and it determined *n*_1_ = 33, *n* = 34 and λ = 8 months with $\hat{\alpha }=0.047$ and $\hat{\beta }=0.196$. The rule implies that an additional patient will be enrolled after the first stage, and it is inappropriate from a practical point of view. Rather than considering two-stage design with a threshold, two-stage design with different thresholds λ_1_ and λ_2_ would be suggested to obtain the reasonable decision rule. Table 5 provides the summary results of the optimal two-stage design, which search *n*_1_, *n*, and λ_2_ for a fixed threshold λ_1_ for the first stage. When survivals are assumed to follow the exponential distribution and threshold for the first stage is 5.5 months, the optimal two-stage design based on median event time test determined *n*_1_ = 17, *n* = 32, λ_2_ = 8.5 months. Specifically, the interim analysis will be performed based on the first 17 enrolled patients by comparing the observed median PFS with 5.5 months. If the observed median PFS is smaller than or equal to 5.5 months, the study is stopped early for futility. Otherwise, additional 15 patients are enrolled to have the total sample size of 32. At the final analysis, the observed median PFS with 32 patients is compared with the threshold 8.5 months, and we claim the addition of bevacizumab to the standard therapy improves PFS if the observed median PFS is larger than the threshold. This trial decision rule yielded power of 80.2%. When the true median PFS is 5 months, the expected sample size for this trial (i.e., EN) is 17.77 and the probability of early stopping (i.e., PET) is 94%. The required sample size and decision rule are changed for the different choice of λ_1_.

**Table 5:** Summary results of optimal two-stage design with a fixed threshold λ_1_ when a maximum sample size *M* is 35.

	*n*_1_	*n*	λ_1_	λ_2_	$\hat{\alpha }$	$\hat{\beta }$	EN_0_	PET_0_
Exponential	17	32	5.5	8.5	0.051	0.198	17.77	0.949
	20	29	6	8.7	0.050	0.201	20.45	0.950
Uniform	16	24	5.5	5.8	0.054	0.202	16.43	0.946
	18	31	6	5.6	0.051	0.200	18.66	0.949
Weibull	15	31	5.5	5.9	0.052	0.202	15.83	0.948
	16	34	6	5.7	0.054	0.204	16.97	0.946

In this trial illustration, PFS was assumed to follow exponential distribution, and the decision rule was identified for the trial under the assumption. We further investigated with the nonexponential assumption. As seen in Table 5, decision rules, especially interim monitoring rules, for exponential versus nonexponential survivals are different, which implies that survival distribution assumption matters to design the clinical trials. In practice, earlier phase trials or experts’ knowledge would be able to provide information of survival distribution for Phase II study. We can borrow the information to identify the appropriate decision rule for the study.

## Discussion

Most existing methods generally assume exponential survival distribution to develop statistical methods or design based on median survival time for convenience [24, 25]. From our simulation studies, we found that operating characteristics of the design depend on the survival distribution, and the decision rule of median event time test is different according to the survival assumption. Type I or II error rate can be inflated when survival distribution is misspecified. It is critical for median event time test to specify survival distribution for robust clinical trial research. The specification of survival distributions can be determined by the relevant pilot study or historical trials.

We have proposed several designs for median event time test: single stage, two stage with single threshold, and two stage with two different thresholds. According to investigators’ interest, study objective, and trial assumption, further simulation investigation may be required to explore more rules. Close collaboration with clinicians as well as statistical practice will guide the better and ethic design for the study.

## Conclusion

We proposed methods to test if there is an improvement of median survivals from a new drug compared to the standard therapy. The proposed median event time test provides the required sample size to control type I and II errors for the hypothesis testing based on the median event time. It also provides a decision rule, which is very simple and straightforward, comparing the observed median survival with the identified threshold by the test. Shiny application and R codes were also built along with the method development so that users can implement easily the hypothesis test based on median event time (https://sites.google.com/view/yeonheepark/software). This approach is extended for the trial with interim at which the study monitors futility. The proposed two-stage design based on median event time test is optimal in that the expected sample size is minimized under the null hypothesis. Early stopping for futility enhances ethics in patient care and expedites the discovery of new therapies. Moreover, our methods would reduce unexpected failures in confirmatory phase after the promising results in Phase II study and improve success rate for drug development.

## Appendix

### A. Proof of theoretical result in methods

**Proof of Theorem 1** Let *F*(*y*) and *f*(*y*) be the cdf and pdf, respectively, of the random variable whose median is *ϕ* (i.e., mean *μ* = *ϕ*/log 2). Then, we have *f*(*y*) = log 2 exp(−*y* log 2/*ϕ*)/*ϕ* and *F*(*y*) = 1 − exp(−*y* log 2/*ϕ*). Suppose that we have a sample of size *n* = 2*k* + 1 for some *k* = 0, 1, 2, …. Then, by Cramér [26], the pdf of the sample median is

$$\begin{array}{ccc} g(m) & = & \left\{n!/\left(k!k!\right)\right\}F{\left(m\right)}^{k}{\left\{1-F\left(m\right)\right\}}^{k}f\left(m\right)\\ & = & \left\{n!\;\text{log}\;2/\left(k!k!\phi \right)\right\}{\left\{1-\exp\left(-m\;\text{log}\;2/\phi \right)\right\}}^{k}\exp\left\{-m\left(k+1\right)\;\text{log}\;2/\phi \right\}. \end{array}$$

We now derive the pdf of the sample median for a sample of size *n* = 2*k* for some *k* = 1, 2, …. When *n* is an even number, the sample median is $\hat{m}=\left(Y_{\left(k\right)}+Y_{\left(k+1\right)}\right)/2$, where *Y*_(*k*)_ denotes the *k*th order statistic of the sample. Note that the joint pdf of *Y*_(*k*)_ and *Y*_(*k*+1)_ is

$$\begin{array}{ccc} f(y_{k},y_{k+1}) & = & cf\left(y_{k}\right)f\left(y_{k+1}\right)F{\left(y_{k}\right)}^{k-1}{\left\{1-F\left(y_{k+1}\right)\right\}}^{n-k-1}\\ & = & \left(c/\mu^{2}\right)\exp\left\{-\left(y_{k}+y_{k+1}\right)/\mu \right\}{\left\{1-\exp\left(-y_{k}/\mu \right)\right\}}^{k-1}\exp\left\{-y_{k+1}\left(n-k-1\right)/\mu \right\}, \end{array}$$

where *c* = *n*!/{(*k* − 1)!(*n* − *k* − 1)!} and *μ* = *ϕ*/log 2, for 0 < *y*_*k*_ < *y*_*k*+1_ < ∞. Let *R* = *Y*_(*k*+1)_ − *Y*_(*k*)_ and *V* = (*Y*_(*k*)_ + *Y*_(*k*+1)_)/2. Then, *V* is our interest and we have an one-to-one transformation from (*Y*_(*k*)_, *Y*_(*k*+1)_) to (*R*, *V*). Since the Jacobian for this transformation is −1, the joint pdf of (*R*, *V*) is

$$\begin{array}{c} f\left(r,v\right)=\left(c/\mu^{2}\right)\exp\left(-2v/\mu \right){\left[1-\exp\left\{\left(v-r/2\right)/\mu \right\}\right]}^{k-1}\exp\left\{-\left(v+r/2\right)\left(n-k-1\right)/\mu \right\}, \end{array}$$

for 0 < *r* < ∞ and *r*/2 < *v* < ∞. Thus, the pdf of the sample median is

$$\begin{array}{ccc} f_{V}\left(v\right) & = & \int_{0}^{2v}f\left(r,v\right)\text{d}r\\ & = & \int_{0}^{2v}c{\left(\;\text{log}\;2/\phi \right)}^{2}\exp\left(-2v\;\text{log}\;2/\phi \right){\left[1-\exp\left\{-\;\text{log}\;2\left(v-r/2\right)/\phi \right\}\right]}^{k-1}\\ & & \times \exp\{-\;\text{log}\;2(v+r/2)(n-k-1)/\phi \}\text{d}r \end{array}$$

for 0 < *v* < ∞.

### B. Median event time test for other parametric survival distributions

We provide two theoretical results stating the distribution function of the sample median event time for uniform and weibull distributions.

**Theorem 2**

1. *Let Y*_1_, …, *Y*_*n*_ *be random variables from a uniform distribution with lower bound parameter a and median ϕ (i.e., upper bound parameter is* 2*ϕ* − *a*). *Then*,
  $$\begin{array}{c} g_{n}\left(m\right)=\frac{n!{\left(m-a\right)}^{k}{\left(2\phi -a-m\right)}^{k}}{k!k!{\left\{2\left(\phi -a\right)\right\}}^{2k+1}} \end{array}$$
  *for n* = 2*k* + 1 *and*
  $$\begin{array}{c} g_{n}\left(m\right)=\{\begin{array}{cc} \int_{0}^{2(m-a)}\frac{c{\left(m-a-r/2\right)}^{k-1}{\left(2\phi -a-m-r/2\right)}^{n-k-1}}{{\left\{2\left(\phi -a\right)\right\}}^{n}}\;dr & \;a\leq m\leq \phi \\ \int_{0}^{-2(m-2\phi +a)}\frac{c{\left(m-a-r/2\right)}^{k-1}{\left(2\phi -a-m-r/2\right)}^{n-k-1}}{{\left\{2\left(\phi -a\right)\right\}}^{n}}\;dr & \;\phi <m\leq 2\phi -a \end{array} \end{array}$$
  *where c* = *n*!/{(*k* − 1)!(*n* − *k* − 1)!}, *for n* = 2*k*.
2. *Let Y*_1_, …, *Y*_*n*_ *be random variables from a uniform distribution with upper bound parameter b and median ϕ (i.e., lower bound parameter is* 2*ϕ* − *b*). *Then*,
  $$\begin{array}{c} g_{n}\left(m\right)=\frac{n!{\left(m-2\phi +b\right)}^{k}{\left(b-m\right)}^{k}}{k!k!{\left\{2\left(b-\phi \right)\right\}}^{2k+1}} \end{array}$$
  *for n* = 2*k* + 1 *and*
  $$\begin{array}{c} g_{n}\left(m\right)=\{\begin{array}{cc} \int_{0}^{2(m-2\phi +b)}\frac{c{\left(m-r/2-2\phi +b\right)}^{k-1}{\left(b-m-r/2\right)}^{n-k-1}}{{\left\{2\left(b-\phi \right)\right\}}^{n}}\;dr & \;2\phi -b\leq m\leq \phi \\ \int_{0}^{2(b-m)}\frac{c{\left(m-r/2-2\phi +b\right)}^{k-1}{\left(b-m-r/2\right)}^{n-k-1}}{{\left\{2\left(b-\phi \right)\right\}}^{n}}\;dr & \;\phi <m\leq b \end{array} \end{array}$$
  *where c* = *n*!/{(*k* − 1)!(*n* − *k* − 1)!}, *for n* = 2*k*.

**Proof**. We first consider a uniform random variable with lower bound parameter *a* and median *ϕ*. Then, we have pdf *f*(*y*) = 1/{2(*ϕ* − *a*)} for *a* ≤ *y* ≤ 2*ϕ* − *a* and cdf

$$\begin{array}{c} F\left(y\right)=\{\begin{array}{cc} 0 & \;y<a\\ \left(y-a)/\{2(\phi -a)\right\} & \;a\leq y\leq 2\phi -a\\ 1 & \;y>2\phi -a \end{array}. \end{array}$$

By Cramér [26], if sample size is *n* = 2*k* + 1 for some *k* = 0, 1, 2, …, the pdf of the sample median is

$$\begin{array}{c} g_{n}\left(m\right)=\frac{n!{\left(m-a\right)}^{k}{\left(2\phi -a-m\right)}^{k}}{k!k!{\left\{2\left(\phi -a\right)\right\}}^{2k+1}}. \end{array}$$

When the sample size is *n* = 2*k* for some *k* = 1, 2, …, we use the joint pdf of *Y*_(*k*)_ and *Y*_(*k*+1)_, where *Y*_(*k*)_ denotes the *k*th order statistic of the sample, given by

$$\begin{array}{c} f\left(y_{k},y_{k+1}\right)=c{\left(y_{k}-a\right)}^{k-1}{\left(2\phi -a-y_{k+1}\right)}^{n-k-1}/{\left\{2\left(\phi -a\right)\right\}}^{n}, \end{array}$$

where *c* = *n*!/{(*k* − 1)!(*n* − *k* − 1)!}, for *a* ≤ *y*_*k*_ < *y*_*k*+1_ ≤ 2*ϕ* − *a*. Let *R* = *Y*_(*k*+1)_ − *Y*_(*k*)_ and *V* = (*Y*_(*k*)_ + *Y*_(*k*+1)_)/2. The one-to-one transformation from (*Y*_(*k*)_, *Y*_(*k*+1)_) to (*R*, *V*) gives the joint pdf of (*R*, *V*) is

$$\begin{array}{c} f\left(r,v\right)=c{\left(v-a-r/2\right)}^{k-1}{\left(2\phi -a-v-r/2\right)}^{n-k-1}/{\left\{2\left(\phi -a\right)\right\}}^{n} \end{array}$$

for 0 < *r* ≤ 2(*ϕ* − *a*) and *r*/2 + *a* ≤ *v* ≤ −*r*/2 + 2*ϕ* − *a*. Thus, the pdf of the sample median is the marginal density of *V*, which is

$$\begin{array}{c} f_{V}\left(v\right)=\{\begin{array}{cc} \int_{0}^{2(v-a)}\frac{c{\left(v-a-r/2\right)}^{k-1}{\left(2\phi -a-v-r/2\right)}^{n-k-1}}{{\left\{2\left(\phi -a\right)\right\}}^{n}}\;dr & \;a\leq v\leq \phi \\ \int_{0}^{-2(v-2\phi +a)}\frac{c{\left(v-a-r/2\right)}^{k-1}{\left(2\phi -a-v-r/2\right)}^{n-k-1}}{{\left\{2\left(\phi -a\right)\right\}}^{n}}\;dr & \;\phi <v\leq 2\phi -a \end{array}. \end{array}$$

Similarly, for a uniform random variable with upper bound parameter *b* and median *ϕ*, we have pdf *f*(*y*) = 1/{2(*b* − *ϕ*)} for 2*ϕ* − *b* ≤ *y* ≤ *b* and cdf

$$\begin{array}{c} F\left(y\right)=\{\begin{array}{cc} 0 & \;y<2\phi -b\\ \left(y-2\phi +b)/\{2(b-\phi )\right\} & \;2\phi -b\leq y\leq b\\ 1 & \;y>b \end{array}. \end{array}$$

It is easy to verify that

$$\begin{array}{c} g_{n}\left(m\right)=\frac{n!{\left(m-2\phi +b\right)}^{k}{\left(b-m\right)}^{k}}{k!k!{\left\{2\left(b-\phi \right)\right\}}^{2k+1}},\;n=2k+1\;\text{for}\;\text{some}\;k=0,1,2,\ldots \end{array}$$

and

$$\begin{array}{c} g_{n}\left(m\right)=\{\begin{array}{cc} \int_{0}^{2(m-2\phi +b)}\frac{c{\left(m-r/2-2\phi +b\right)}^{k-1}{\left(b-m-r/2\right)}^{n-k-1}}{{\left\{2\left(b-\phi \right)\right\}}^{n}}\;dr & \;2\phi -b\leq m\leq \phi \\ \int_{0}^{2(b-m)}\frac{c{\left(m-r/2-2\phi +b\right)}^{k-1}{\left(b-m-r/2\right)}^{n-k-1}}{{\left\{2\left(b-\phi \right)\right\}}^{n}}\;dr & \;\phi <m\leq b \end{array}, \end{array}$$

*n* = 2*k* for some *k* = 1, 2, ….

**Theorem 3** *Let Y*_1_, …, *Y*_*n*_ *be random variables from a weibull distribution with shape parameter τ and median ϕ (i.e., scale parameter is ϕ*/(log 2)^1/*τ*^). *Then*,

$$\begin{array}{ccc} g_{n}\left(m\right) & = & \frac{n!\tau {\left(\;\text{log}\;2\right)}^{1/\tau }}{k!k!\phi }{\left\{{\left(\;\text{log}\;2\right)}^{1/\tau }m/\phi \right\}}^{\tau -1}{\left(1-\exp[-{\left\{{\left(\;\text{log}\;2\right)}^{1/\tau }m/\phi \right\}}^{\tau }]\right)}^{k}\\ & & \times \exp[-{\left\{{\left(\;\text{log}\;2\right)}^{1/\tau }m/\phi \right\}}^{\tau }\left(k+1\right)] \end{array}$$

*for n* = 2*k* + 1 *and*

$$\begin{array}{ccc} g_{n}\left(m\right) & = & \int_{0}^{2m}c{\left(\tau \;\text{log}\;2/\phi^{\tau }\right)}^{2}{\left\{\left(m-r/2\right)\left(m+r/2\right)\right\}}^{\tau -1}(1-\\ & & \exp\left[-{\left\{{\left(\;\text{log}\;2\right)}^{1/\tau }\left(m-r/2\right)/\phi \right\}}^{\tau }\right]{)}^{k-1}\exp[-{\left\{{\left(\;\text{log}\;2\right)}^{1/\tau }\left(m-r/2\right)/\phi \right\}}^{\tau }-\\ & & {\left\{{\left(\;\text{log}\;2\right)}^{1/\tau }\left(m+r/2\right)/\phi \right\}}^{\tau }\left(n-k\right)]\;dr, \end{array}$$

*where c* = *n*!/{(*k* − 1)!(*n* − *k* − 1)!}, *for n* = 2*k*.

**Proof**. Let *Y* be a weibull random variable with scale parameter *τ* and median *ϕ*. Then, we have pdf *f*(*y*) = *τ*(log 2)^1/*τ*^{(log 2)^1/*τ*^ *y*/*ϕ*}^*τ*−1^ exp[−{(log 2)^1/*τ*^ *y*/*ϕ*}^*τ*^]/*ϕ* and cdf *F*(*y*) = 1 − exp[−{(log 2)^1/*τ*^ *y*/*ϕ*}^*τ*^]. By Cramér [26], if sample size is *n* = 2*k* + 1 for some *k* = 0, 1, 2, …, the pdf of the sample median is

$$\begin{array}{ccc} g_{n}\left(m\right) & = & \frac{n!\tau {\left(\;\text{log}\;2\right)}^{1/\tau }}{k!k!\phi }{\left\{{\left(\;\text{log}\;2\right)}^{1/\tau }m/\phi \right\}}^{\tau -1}{\left(1-\exp[-{\left\{{\left(\;\text{log}\;2\right)}^{1/\tau }m/\phi \right\}}^{\tau }]\right)}^{k}\\ & & \times \exp[-{\left\{{\left(\;\text{log}\;2\right)}^{1/\tau }m/\phi \right\}}^{\tau }\left(k+1\right)]. \end{array}$$

When the sample size is *n* = 2*k* for some *k* = 1, 2, …, by proof of Theorem 1, the pdf of the sample median is

$$\begin{array}{ccc} g_{n}\left(m\right) & = & \int_{0}^{2m}c{\left(\tau \;\text{log}\;2/\phi^{\tau }\right)}^{2}{\left\{\left(m-r/2\right)\left(m+r/2\right)\right\}}^{\tau -1}(1-\\ & & \exp\left[-{\left\{{\left(\;\text{log}\;2\right)}^{1/\tau }\left(m-r/2\right)/\phi \right\}}^{\tau }\right]{)}^{k-1}\exp[-{\left\{{\left(\;\text{log}\;2\right)}^{1/\tau }\left(m-r/2\right)/\phi \right\}}^{\tau }-\\ & & {\left\{{\left(\;\text{log}\;2\right)}^{1/\tau }\left(m+r/2\right)/\phi \right\}}^{\tau }\left(n-k\right)]\;dr, \end{array}$$

where *c* = *n*!/{(*k* − 1)!(*n* − *k* − 1)!}, for 0 < *m* < ∞.

## Supporting information

S1 File (PDF)
