# Supplementary material for: Optimal two-stage design of single arm Phase II clinical trials based on median event time test
Source: PLoS One. 2021 Feb 8;16(2):e0246448. doi: 10.1371/journal.pone.0246448 (PMC7870013; doi:10.1371/journal.pone.0246448)
Supplement: S1 File — (PDF) [file pone.0246448.s001.pdf]

Supporting Information file for “Optimal Two-Stage Design of Single arm Phase II Clinical Trials based on Median Event Time Test”

Yeonhee Park*

Department of Biostatistics and Medical Informatics, University of Wisconsin, Madison, WI, USA

* ypark56@wisc.edu

We provide R codes to implement the methods for median event time test when survivals are assumed to follow exponential distribution. Main functions are “calc.mett” and “METT2E” for one-stage and two-stage optimal designs, respectively. The followings describe arguments for the functions.

- alpha = Target type I error rate
- beta = Target type II error rate
- phi.0 or t.n = Null median event time
- phi.a or t.a = Alternative median event time
- M = Maximum sample size for the study
- rate = Patient accrual rate per month
- FUP = Follow-up time (months)
- nsim = Number of simulations
- nincm = Increment of the sequence for sample size to be searched
- lamincm = Increment of the sequence for lambda to be searched
- eps1 = Maximum acceptable difference between the estimated type I error rate from the trial design and the target error rate (alpha), epsilon1
- eps2 = Maximum acceptable difference between the estimated type I error rate from the trial design and the target error rate (beta), epsilon2
- n1init = Initial value of n1 to be searched
- n1last = Last value of n1 to be searched
- seed =seed number

```
library("pracma")
library("survival")
library("survminer")

ahatfct <- function(n, lam, phi.0){
  k <- n/2
  phi <- phi.0
```

```
  integrand <- function(t,r) {
    (log(2)/phi)^2*((1-exp(-(t-r/2)*log(2)/phi))^(k-1))*
      exp(-t*2*log(2)/phi)*exp(-log(2)*(t+r/2)*(n-k-1)/phi)*factorial(n)/
      (factorial(k-1)*factorial(n-k-1))
  }
  fmin <- function(t) 2*t
  res <- integral2(integrand, 0, lam, 0, fmin,
                   maxlist=300, reltol = 1e-3)
  ahat <- 1-res$Q
  ahat
}

bhatfct <- function(n, lam, phi.a){
  k <- n/2
  phi <- phi.a
  integrand <- function(t,r) {
    (log(2)/phi)^2*((1-exp(-(t-r/2)*log(2)/phi))^(k-1))*
      exp(-t*2*log(2)/phi)*exp(-log(2)*(t+r/2)*(n-k-1)/phi)*factorial(n)/
      (factorial(k-1)*factorial(n-k-1))
  }
  fmin <- function(t) 2*t
  res <- integral2(integrand, 0, lam, 0, fmin,
                   maxlist=300, reltol = 1e-3)
  bhat <- res$Q
  bhat
}

ahatfctodd <- function(n, lam, phi.0){
  k <- (n-1)/2
  phi <- phi.0

  integrand <- function(x) {
    (1-exp(-x*log(2)/phi))^k*exp(-x*(k+1)*log(2)/phi)
  }

  res <- integrate(integrand, lower = 0, upper = lam)
  ahat <- 1-factorial(n)*log(2)*res$value/(factorial(k)*factorial(k)*phi)
  ahat
}

bhatfctodd <- function(n, lam, phi.a){
  k <- (n-1)/2
  phi <- phi.a
  integrand <- function(x) {
    (1-exp(-x*log(2)/phi))^k*exp(-x*(k+1)*log(2)/phi)
  }
  res <- integrate(integrand, lower = 0, upper = lam)
  bhat <- factorial(n)*log(2)*res$value/(factorial(k)*factorial(k)*phi)
  bhat
}

calc.mett <- function(alpha, beta, phi.0, phi.a, M){
  pp <- seq(3, M, by=1)
  qq <- seq(phi.0, phi.a, by=0.1)
  llp <-length(pp)
  ll <- length(qq)
  ahat=phat <- matrix(rep(0, ll*llp), ncol=ll)
```

```
  for(i in 1:llp){
    for(t in 1:ll){
      n <- pp[i]
      lam <- qq[t]
      if((n%%2)==0){
        ahat[i,t] <- ahatfct(n, lam, phi.0)
        phat[i,t] <- bhatfct(n, lam, phi.a)
      }else{
        ahat[i,t] <- ahatfctodd(n, lam, phi.0)
        phat[i,t] <- bhatfctodd(n, lam, phi.a)
      }
    }
  }
  ff <- (ahat-alpha)^2+(phat-beta)^2
  ind <- which(ff == min(ff), arr.ind = TRUE)
  i1<-ind[1]
  i2<-ind[2]
  n <- pp[i1]
  lambda <- qq[i2]
  alphahat <- round(ahat[i1, i2], 4)
  betahat <- round(phat[i1, i2], 4)
  res <- list(n, lambda, alphahat, betahat)
  names(res) <- c("n", "lambda", "alphahat", "betahat")
  return(res)
}

opres.exp <- function(t.n, t.a, mu_true, lam, n.interim, rate, FUP, nsim){
  nmax = max(n.interim)
  nobs = n.interim+1
  nobs[length(nobs)] = nmax
  out1 <- c()
  pts=ttrial <- c()
  for (sim in 1:nsim){
    wait.t = rexp(nmax,rate = rate)
    arrival.t = cumsum(wait.t)
    event.t = rexp(nmax,rate=log(2)/mu_true)
    tobs = arrival.t[nobs]
    tobs[length(tobs)] = tobs[length(tobs)] + FUP
    k=1
    n.fail = sum(arrival.t[1:n.interim[k]] + event.t[1:n.interim[k]] <= tobs[k])
    t.event = rep(0,n.interim[k])
    t.ind = rep(0,n.interim[k])
    for(j in 1:length(t.event)) {
      t.event[j] = ifelse(arrival.t[j]+event.t[j]<=tobs[k],event.t[j],
      tobs[k]-arrival.t[j])
      t.ind[j] = ifelse(arrival.t[j]+event.t[j]<=tobs[k],1,0)
    }
    fit <- survfit(Surv(t.event, t.ind)~1)
    if(min(fit$surv)>0.5){
      phihat <- max(t.event)
    }else{
      phihat <- summary(fit)$table[5]
    }
    if(phihat <= lam){
      out1[sim] <- 21
      pts[sim] <- n.interim[k]
      ttrial[sim] <- tobs[k]
```

```
    }else{
      k=2
      n.fail = sum(arrival.t[1:n.interim[k]] + event.t[1:n.interim[k]] <= tobs[k])
      t.event = rep(0,n.interim[k])
      t.ind = rep(0,n.interim[k])
      for(j in 1:length(t.event)) {
        t.event[j] = ifelse(arrival.t[j]+event.t[j]<=tobs[k],event.t[j],
        tobs[k]-arrival.t[j])
        t.ind[j] = ifelse(arrival.t[j]+event.t[j]<=tobs[k],1,0)
      }
      fit <- survfit(Surv(t.event, t.ind)~1)
      if(min(fit$surv)>0.5){
        phihat <- max(t.event)
      }else{
        phihat <- summary(fit)$table[5]
      }
      if(phihat > lam){
        out1[sim] <- 1
        pts[sim] <- n.interim[k]
        ttrial[sim] <- tobs[k]
      }else{
        out1[sim] <- 21
        pts[sim] <- n.interim[k]
        ttrial[sim] <- tobs[k]
      }
    }
  }
  phat <- length(which(out1==1))/nsim
  earlystop <- length(which(out1==21))/nsim
  mpts <- mean(pts)
  mtrial <- mean(ttrial)
  res <-list(phat, earlystop, mpts, mtrial)
  names(res) <- c("phat", "earlystop", "mpts", "mtrial")
  return(res)
}

n1init.exp <- function(init0, M, t.n, t.a, rate, FUP, nsim, alpha, beta){
  init00 <- init0 + 1
  pp <- seq(init00, M, by=5)
  llp <-length(pp)
  qq <- seq(t.n, t.a, by=0.2)
  ll <- length(qq)
  ahat=phat=PET <- matrix(rep(10000, ll*llp), ncol=ll)

  for(i in 1:llp){
    nval <- pp[i]
    n.interim = c(init0, nval)
    for(t in 1:ll){
      lamphi <- qq[t]
      out1 <- opres.exp(t.n, t.a, t.n, lam=lamphi, n.interim, rate, FUP, nsim)
      ahat[i,t] <- out1$phat
      out2 <- opres.exp(t.n, t.a, t.a, lam=lamphi, n.interim, rate, FUP, nsim)
      phat[i,t] <- 1-out2$phat
    }
  }
  ff <- (ahat-alpha)^2+(phat-beta)^2
  ind <- which(ff == min(ff), arr.ind = TRUE)
```

```
  i1<-ind[1]
  i2<-ind[2]
  alphahat <- ahat[i1, i2]
  betahat <- phat[i1, i2]
  res <-list(betahat, alphahat)
  names(res) <- c("betahat", "alphahat")
  return(res)
}

METT2E <- function(alpha, beta, M, t.n, t.a, rate, FUP, nsim,
                   nincm, lamincm, eps1, eps2, n1init, n1last, seed){

  if(is.null(n1init)==TRUE){
    n10 <- floor(M/2)
    n1res1 <- n1init.exp(n10, M, t.n, t.a, rate, FUP, nsim=1000, alpha, beta)
    if(abs(n1res1$alphahat-alpha)>eps1 & abs(n1res1$betahat-beta)>eps2) {
      n1init <- n10
    }else{
      n11 <- floor(M/4)
      n1res2 <- n1init.exp(n11, M, t.n, t.a, rate, FUP, nsim=1000, alpha, beta)
      if(abs(n1res2$alphahat-alpha)>eps1 & abs(n1res2$betahat-beta)>eps2) {
        n1init <- n11
      }else{
        n1init <- 3
      }
    }
  }
  if(is.null(n1last)==TRUE){
    n1last <- M-1
  }
  if(is.null(seed)==TRUE){
    seed <- 840130
  }
  n1 <- seq(n1init, n1last, by=1)
  n1l <- length(n1)
  n.out = lam.out <- c()
  PETn = EN =alphahat = betahat <- c()
  inderr <- rep(0, n1l)
  for(k in 1:n1l){
    cat(k, "of", n1l, ": n1 =", n1[k], "\n")
    aaa <- seed + k
    set.seed(aaa)
    pp <- seq(n1[k] + 1, M, by=nincm)
    llp <-length(pp)
    qq <- seq(t.n, t.a, by=lamincm)
    ll <- length(qq)
    ahat=phat=PET <- matrix(rep(10000, ll*llp), ncol=ll)

    for(i in 1:llp){
      nval <- pp[i]
      for(t in 1:ll){
        lamphi <- qq[t]
        n.interim = c(n1[k], nval)
        out1 <- opres.exp(t.n, t.a, t.n, lam=lamphi, n.interim, rate, FUP, nsim)
        ahat[i,t] <- out1$phat
        out2 <- opres.exp(t.n, t.a, t.a, lam=lamphi, n.interim, rate, FUP, nsim)
        phat[i,t] <- 1-out2$phat
```

```
        PET[i,t] <- out1$earlystop
      }
    }

    ff <- (ahat-alpha)^2+(phat-beta)^2
    ind <- which(ff == min(ff), arr.ind = TRUE)
    if(length(ind)>2){
      i1<-ind[1,1]
      i2<-ind[1,2]
    }else{
      i1<-ind[1]
      i2<-ind[2]
    }
    n.out[k] <- pp[i1]
    lam.out[k] <- qq[i2]
    PETn[k] <- PET[i1,i2]
    EN[k] <- n1[k]+(1-PETn[k])*(n.out[k]-n1[k])
    alphahat[k] <- ahat[i1, i2]
    betahat[k] <- phat[i1, i2]
    if (abs(ahat[i1, i2]-alpha)<eps1 & abs(phat[i1, i2]-beta)<eps2) {
      inderr[k]<-1
      cat("When n1=", n1[k], "n=", n.out[k], "and lambda=", lam.out[k],
          "Then alphahat is ", ahat[i1, i2], "and betahat is ", phat[i1, i2],
          "EN=", EN[k], "\n")
    }else{
      cat("Warning: Rule is not identified for target error rates", alpha,
      "and", beta, "\n")
    }
  }
  llinderr <- length(which(inderr==1))
  if(llinderr==0){
    cat("---------- Change values of n1init and n1last ----------", "\n")
    n1res= nres= lamres= enres= petres= ares= bres <- NA

  }else{
    EN1 <- EN[which(inderr==1)]
    enres <- min(EN1)
    n11 <- n1[which(inderr==1)]
    n1res <- n11[which(EN1==min(EN1))]
    nres1 <- n.out[which(inderr==1)]
    nres <- nres1[which(EN1==min(EN1))]
    lamres1 <- lam.out[which(inderr==1)]
    lamres <- lamres1[which(EN1==min(EN1))]
    PETn1 <- PETn[which(inderr==1)]
    petres <- PETn1[which(EN1==min(EN1))]
    ares1 <- alphahat[which(inderr==1)]
    ares <- ares1[which(EN1==min(EN1))]
    bres1 <- betahat[which(inderr==1)]
    bres <- bres1[which(EN1==min(EN1))]
  }

  res <- list(n1res, nres, lamres, enres, petres, ares, bres)
  names(res) <- c("n1", "n", "lambda", "EN", "PET0", "alphahat", "betahat")
  return(res)
}
```
